# Dynamic markers based on blood perfusion fluctuations for selecting skin melanocytic lesions for biopsy

**DOI:** 10.1038/srep12825

**Published:** 2015-08-11

**Authors:** Gemma Lancaster, Aneta Stefanovska, Margherita Pesce, Gian Marco Vezzoni, Barbara Loggini, Raffaele Pingitore, Fabrizio Ghiara, Paolo Barachini, Gregorio Cervadoro, Marco Romanelli, Marco Rossi

**Affiliations:** 1 Department of Physics, Lancaster University, UK; 2 Department of Clinical and Experimental Medicine, University of Pisa, Italy; 3 Unit 3 of Pathological Anatomy and Histology, Hospital of Pisa, Italy; 4 Department of Translational Research and New Technologies in Medicine, Pisa University, Italy; 5 Dermatology Unit, Hospital of Pisa, Italy

## Abstract

Skin malignant melanoma is a highly angiogenic cancer, necessitating early diagnosis for positive prognosis. The current diagnostic standard of biopsy and histological examination inevitably leads to many unnecessary invasive excisions. Here, we propose a non-invasive method of identification of melanoma based on blood flow dynamics. We consider a wide frequency range from 0.005–2 Hz associated with both local vascular regulation and effects of cardiac pulsation. Combining uniquely the power of oscillations associated with individual physiological processes we obtain a marker which distinguishes between melanoma and atypical nevi with sensitivity of 100% and specificity of 90.9%. The method reveals valuable functional information about the melanoma microenvironment. It also provides the means for simple, accurate, *in vivo* distinction between malignant melanoma and atypical nevi, and may lead to a substantial reduction in the number of biopsies currently undertaken.

As the most deadly skin cancer, skin malignant melanoma (SMM) is responsible for 75% of all skin cancer related deaths^1^, whilst accounting for only a small proportion of skin cancer incidence. SMM is an aggressive cancer which shows resistance to many conventional cancer treatments. Therefore, emphasis remains on early diagnosis for a positive long term prognosis.

Despite the development of a large number of non-invasive alternatives^2^,^3^,^4^,^5^,^6^,^7^,^8^,^9^, the current gold-standard in melanoma diagnosis remains as the examination of a skin lesion by a trained dermatologist, followed by histological examination of an invasive excisional biopsy of the skin specimen^2^,^3^,^8^,^9^. This method results in sensitivity and specificity ranging from 65–80%^9^. Dermoscopy, a non-invasive, *in vivo* examination based on microscopy, improves diagnostic accuracy of SMM compared to inspection by the unaided eye^2^, but this accuracy strongly depends upon the expertise of the examiner^3^,^4^. The relatively low specificity of diagnosis resulting from these methods inevitably leads to many unnecessary biopsies, and thus to discomfort and distress to patients. Therefore, the diagnosis of early stage SMM through non-invasive techniques remains an active area of research.

Angiogenesis is a common feature of SMM, and plays a key role in tumour growth and metastasis^10^. SMM vasculature formed via angiogenesis is characterized by tufted, glomerulus-like capillaries with hypervascularization and growth toward the tumour. These morphological characteristics of SMM-associated blood vessels may affect SMM blood perfusion.

Blood perfusion in skin cancer has previously been investigated using laser Doppler flowmetry (LDF)^11^,^12^,^13^,^14^,^15^, a non-invasive technique which has been applied successfully to the characterisation of blood perfusion and its dynamics in various vascular diseases^16^, as well as during angiogenesis^17^. Skin cancers from basal cell carcinoma to malignant melanoma have been described in terms of their average blood perfusion values in these studies^12^,^13^,^14^,^15^; however, the important dynamical properties of their perfusion have been largely overlooked.

The LDF signal is obtained as a difference between emitted light and back-scattered light that has been Doppler-shifted by the complex movement of light-reflecting particles (mainly red blood cells). This movement corresponds to the continuous circulation of the blood through the microvascular bed; the diffusion into neighbouring cells of substances being brought by the blood is known as blood perfusion. In this manuscript we will therefore use ‘blood flow’ and ‘blood perfusion’ almost interchangeably.

Traditionally, the fluctuations present in blood flow signals have been considered as a source of irreproducibility, arising from stochastic processes. Contrary to this, by combining the examination of much longer time series and the use of wavelet analysis, patterns consistent with determinism in these systems have been obtained from the analysis of blood flow signals^18^,^19^. Using these methods, specific physiological processes over time can be followed, revealing a large amount of data which is lost through averaging^18^,^20^,^21^.

The heterogeneity of the skin microvasculature, which is exacerbated further in the presence of a tumour, means that temporal variations in perfusion must be taken into account in order to fully represent microvascular flow. Previous studies^18^,^20^,^21^,^22^ into microvascular blood flow dynamics in healthy human skin have revealed six distinct oscillatory components, attributable to different physiological functions: interval I (0.6–2 Hz) related to cardiac activity, interval II (0.145–0.6 Hz) related to respiratory activity, interval III (0.052–0.145 Hz) related to microvessel smooth muscle cell activity, interval IV (0.021–0.052 Hz) related to microvessel innervation^23^ and intervals V & VI (0.0095–0.021 Hz and 0.005–0.0095 Hz, respectively) related to endothelial activity, both nitric oxide (NO) dependent and independent^21^. Using LDF, changes in these oscillations can be non-invasively observed, providing information regarding real physiological processes *in vivo,* including the ability of blood vessels to rhythmically change their diameter, known as vasomotion^24^.

Here, we tested the hypothesis that the examination of blood flow dynamics in skin atypical nevi could facilitate the non-invasive identification of skin malignant melanoma. A secondary hypothesis was that the same examination would provide increased understanding of microcirculatory pathophysiology in SMM. To test these hypotheses, skin blood perfusion was recorded and analyzed in terms of its dynamics in SMM, atypical nevi, typical benign nevi and psoriasis lesions using LDF and wavelet analysis.

## Results

### Subjects

Fifty five subjects with a clinically atypical skin nevus were enrolled in the study, according to the inclusion criteria. Thirty clinically healthy volunteers with clinically typical skin nevi and nine patients with psoriasis were also enrolled in the study. Five of the enrolled subjects (one with clinically atypical nevus, which was diagnosed as SMM at histological examination, and four with clinically typical nevus) were excluded from the final results as their LDF tracings contained anomalous spikes, a likely erroneous optical effect.

### Histological examination

On histological examination, the 54 clinically atypical nevi included in the final data resulted in skin malignant melanoma in 10 cases, benign atypical nevi in 33 cases and benign typical nevi in 11 cases (for further details see Tables 1 & 2). Atypical nevi were identified as compound, junctional and dermal nevi. A wide range of dysplasia, from slight to severe, was studied (see Table 3).

**Figure 1:**
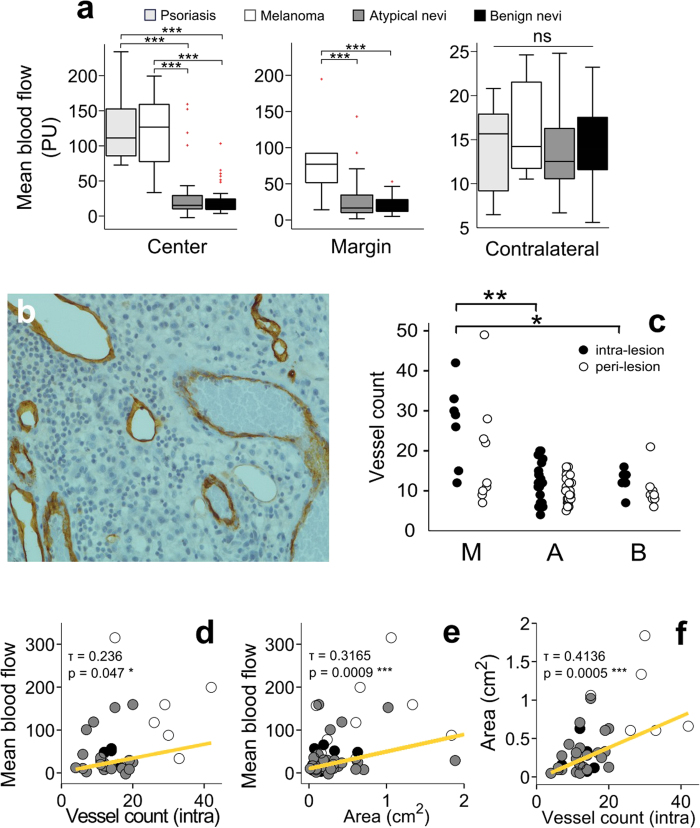
(**a**) Mean blood flow values for all recording locations and groups of studied lesions. Data are presented as boxplots where the upper and lower limits of each box represent the 75^th^ and 25^th^ percentiles, respectively; the line between these is the median value. Outliers are shown in red. (**b**) Intra-lesion micro-vessels highlighted with CD34 Mab (Ventan Medical System) at the level of a skin malignant melanoma. (**c**) Results of micro-vessel count for all examined groups. (**d**–**f**) show the correlation found for all malignant melanomas studied between vessel count and mean blood flow detected at the level of the lesion center (**d**), between lesion area and mean blood flow detected at the level of the lesion center (**e**), and between vessel count and lesion area (**f**). *p < 0.05; ***p < 0.001; PU = perfusion unit.

The 11 clinically atypical nevi which were revealed to be benign typical nevi during histological examination were considered to belong to the atypical nevi group for the purposes of diagnosis, but to the benign typical nevi group for the purposes of blood flow dynamics analysis. Results of microvessel examination are reported in Fig. 1. SMMs had a significantly higher number of intra-lesion microvessels when compared to atypical nevi, but did not significantly differ from atypical nevi in the number of peri-lesional vessels.

### Average blood perfusion values

Results of average blood flow values are reported in Table 4 and Fig. 1. The SMM group showed significantly higher average blood flow values (*p* = 0.0000, *p* = 0.0004, respectively) at lesion centers and margins [126.8 PU (80.0–158.6) and 77.1 PU (53.4–91.8), respectively] compared to both the histologically atypical nevi [15.2 PU (10.1–29.1) and 16.6 PU (10.4–32.5), respectively] and the benign typical nevi [18.6 PU (9.5–23.9) and 19.1 PU (12.2–28.0), respectively]. No significant difference in mean blood flow values was observed between the 10 SMM and the 9 psoriasis lesions studied [111.3 PU (85.8–125.6)], at the level of the lesion center.

SMM, histologically atypical nevi and psoriasis lesions all showed significantly higher mean blood flow values at their lesion centers (*p* = 0.0020, *p* = 0.0179 and *p* = 0.0039, respectively), compared to the contralateral skin site of the same subject [14.2 PU (11.8–20.6), 12.5 PU (10.8–16.0) and 15.7 PU (9.8–17.5), respectively], whilst benign typical nevi did not. No significant differences were found between any groups in mean blood flow values recorded at contralateral healthy skin sites.

### Correlation between histological findings and average blood perfusion values

A significant positive correlation (*p* = 0.047) was observed between mean blood flow values recorded at lesion centers and intra-lesional vessel density in all histologically examined nevi (Fig. 1). No correlation was found between the mean blood flow recorded at lesion margins and the peri-lesional vessel density in the same nevi. A negative correlation (*p* = 0.0128) was found between intra-lesional vessel density and normalized spectral power in interval III at lesion centres.

### Blood perfusion fluctuations

**Figure 2:**
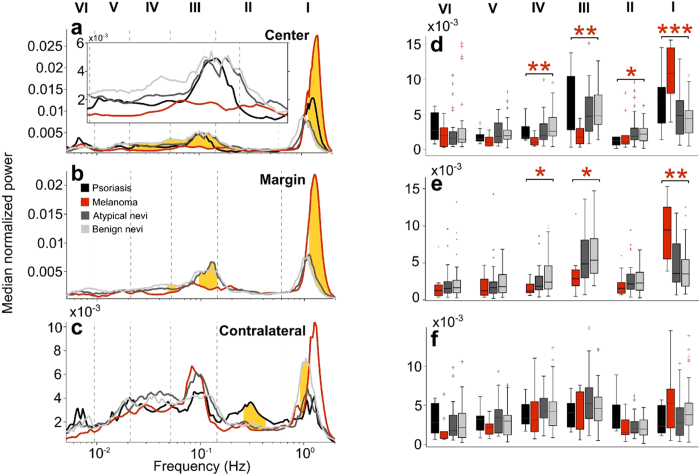
(**a**–**c**) Normalized median spectral power values obtained by wavelet analysis of laser Doppler tracings recorded for 30 minutes in SMM lesions (red line), atypical nevi (dark grey line), benign nevi (light grey line) and psoriasis (black line) in lesions centers (**a**), margins (**b**) and in contralateral healthy skin (**c**). Significant differences are highlighted in yellow (*p* < 0.05) as determined by the Kruskal Wallis test. (**d**–**f**) Normalized spectral power values within the six frequency intervals considered (see text) in SMM (red), atypical nevi (dark grey), benign nevi (light grey) and psoriasis (black) in lesion centers (**d**), margins (**e**) and healthy contralateral skin (**f**). *p < 0.05; **p < 0.01; ***p < 0.001.

Quantitative analysis of blood perfusion fluctuations are summarised in Table 4 and Fig. 2. A significantly lower normalized spectral power in the frequency intervals associated with myogenic (III) and neurogenic (IV) activity (*p* = 0.0006 and *p* = 0.0005, respectively) was observed at the lesion center of SMMs [1.7 (0.9–2.7) and 1.0 (0.6–1.7), respectively] when compared to both histologically atypical nevi [4.7 (2.6–7.5) and 1.9 (1.4–3.5), respectively] and benign nevi [4.8 (3.6–7.6) and 2.6 (1.9–4.3), respectively]. A significantly higher normalized spectral power in the frequency interval associated with cardiac activity (I) (*p* = 0.0003) was observed at the lesion center of SMMs [10.8 (8.2–14.3)] when compared to both histologically atypical nevi [4.9 (2.2–7.3)] and benign nevi [4.4 (2.4–5.5)]. On the contrary, SMMs did not significantly differ from both histologically atypical and benign nevi for the spectral power in the frequency intervals V and VI, associated with endothelial activity.

A significantly lower normalized spectral power in the frequency intervals III & IV (*p* = 0.0066 and *p* = 0.0229, respectively) was observed at lesion margins of SMMs [2.8 (2.0–4.0) and 1.2 (1.0–2.0), respectively] compared to benign nevi [5.3 (3.6–8.2) and 2.4 (1.5–4.5), respectively], but only in interval IV when compared to atypical nevi (*p* = 0.0457) [1.8 (1.3–3.1)]. As was the case for lesion centers, SMMs at margins showed significantly higher (*p* = 0.0034) normalized spectral power in interval I [9.4 (5.8–12.2)] than both atypical nevi [3.6 (2.4–6.7)] and benign nevi [3.5 (1.9–5.4)].

SMMs also showed significantly lower normalized spectral power in the frequency intervals associated with III (myogenic), IV (neurogenic) and V (NO-dependent endothelial activity) frequency intervals, when compared to contralateral skin in the same subjects [5.7 (2.1–6.7), 3.1 (1.8–5.1) and 2.2 (1.4–2.5), respectively) ] (*p* = 0.0020, *p* = 0.0020 and *p* = 0.0039, respectively), whilst exhibiting a higher normalized spectral power associated with I (cardiac) frequency interval [5.2 (2.7–7.1), *p* = 0.002]. The same comparison in atypical nevi revealed significantly lower spectral power in intervals IV [1.9 (1.4–3.5) vs 3.9 (3.3–5.7), *p* = 0.0001] and V [1.8 (1.0–3.7) vs 3.4 (1.5–4.4), *p* = 0.0049], as well as a significantly higher spectral power in interval I [4.9 (2.2–7.3) vs 2.8 (1.2–4.8), *p* = 0.0004], in lesion centers. In contrast, center and contralateral spectral powers only significantly differed in interval IV (center significantly lower) in typical benign nevi, and psoriasis differed only in intervals I (center significantly higher), as well as in the interval associated with respiration, II, (center significantly lower), when compared to contralateral skin in the same subjects.

Comparison of centrally recorded normalized spectral power between atypical and benign nevi revealed no significant differences in any interval except the neurogenic interval IV, while no differences were found during the same comparison for data recorded at margins.

### Distinguishing between skin malignant melanoma and atypical nevi

For the purposes of a diagnostic test, i.e. a dynamical biomarker, the most discriminatory significant differences emerged in the form of three variables, in melanoma:

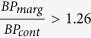
, where BP_marg_ and BP_cont_ are mean blood flows at lesion margins and contralateral skin, respectively,
2. normalized spectral power of cardiac interval I at the lesion margin > 0.0038, and
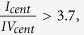
where I_cent_ and IV_cent_ are the total spectral powers in the cardiac and neurogenic frequency intervals, respectively.

**Figure 3:**
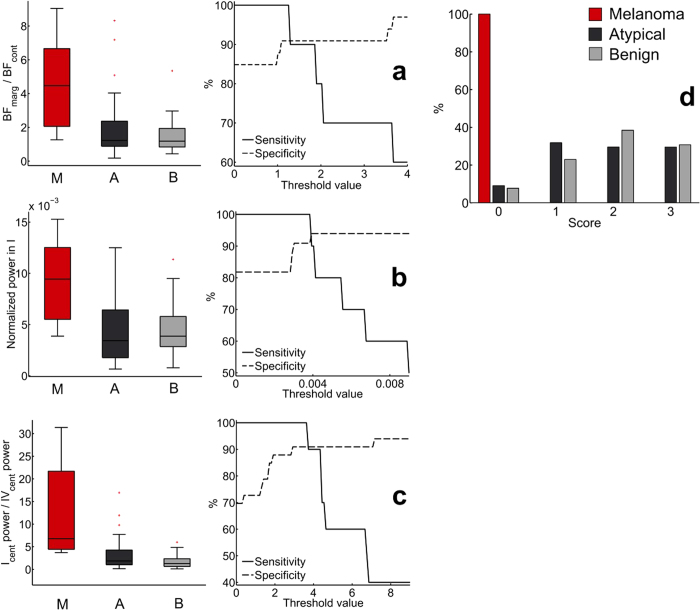
(**a–c**) – boxplots of the three values used in the diagnostic test (left, p values 0.0009, 0.0035 & 0.0001, respectively) and sensitivities and specificities with varying thresholds (right). (**d**) Scores obtained from the diagnostic test (where 0 = melanoma), based on threshold values of 1.26, 0.0038 and 3.7.

Combining these characteristics results in a sensitivity of 100% and a specificity of 90.9% in discriminating between SMMs and atypical nevi, based on the available data (positive predictive value = 62.5%, negative predictive value = 100%), see Fig. 3.

## Discussion

Research into non-invasive diagnostic methods for SMM has yielded many techniques in recent years^1^,^2^,^3^,^4^,^5^,^6^,^7^, none of which has been transferred into routine clinical practice. Disadvantages of such methods usually lie in their subjective nature; for example, any technique which relies on imaging still requires extensive training, and any resulting diagnoses may vary between practitioners. The main disadvantage however, is the inadequate sensitivity^1^,^2^,^3^,^4^,^5^,^6^,^7^, which is intrinsic to many of these methods, leading to a situation where unnecessary biopsies are still performed due to the false negatives that these techniques may produce.

Here we present a method of distinguishing between SMM and atypical nevi, one of the most problematic differential diagnoses in clinical practice. This is due to very similar appearance of these two kinds of skin lesions; with many atypical nevi failing the initial ABCD test. Unlike previous studies based on LDF^13^,^14^,^15^,^25^, which took into account only average perfusion values, we quantified the inherent fluctuations across a wide range of frequencies in SMMs, atypical nevi, benign nevi and psoriasis, using wavelet analysis. This time-frequency representation is of even greater importance in SMM vasculature than in normal skin, due to the heterogeneity introduced by tumour angiogenesis. Häfner *et al.*^12^ also considered the frequency content of LDF signals in SMM, but the recording time was only 3.3 minutes. In the current study we collected blood flow data for a period of 30 minutes. This allowed us to observe significant differences in blood perfusion fluctuations at lower frequencies that were not previously examined. In particular, among the lower frequencies, the neurogenic frequency interval showed the greatest difference, and forms the basis of one of our biomarkers.

By combining dynamical biomarkers, based on significant differences in blood perfusion and its fluctuations, we obtained a binary result, melanoma or non-melanoma, with a sensitivity of 100% and a specificity of 90.9%, based on the available data from clinically atypical nevi. Although it is likely that the exact cut off values obtained for each of the three tests may vary slightly with further recruitment of subjects, we are confident that the main differences in blood flow dynamics on which the tests are based will remain, based on statistical power testing (see supplementary material). We confirmed the previously obtained result of increased blood perfusion in melanoma, when compared with atypical lesions and healthy skin. Mean flows in healthy contralateral skin did not differ significantly between any group, suggesting that our findings provide information relating to the tumour. However, although promising, this result requires further investigation in a larger, more uniform, data set before it can be recommended as a pre-excision biomarker for selecting skin melanocytic lesions that should be histologically examined.

SMMs also exhibited a significantly lower normalized spectral power in the frequency intervals related to myogenic-, neurogenic- and NO-dependent endothelial vasomotion as compared to the contralateral skin in the same subject, a finding which was also observed, although with the exclusion of the myogenic dependent vasomotion, in atypical nevi. This similarity in blood perfusion dynamics between SMMs and atypical nevi (skin melanocytic lesions which are well known to be more prone to development into SMM than typical benign nevi) suggests that the transformation from atypical skin nevus to SMM is a gradual process, at least regarding the mechanisms involved in blood perfusion fluctuations. Interestingly, a negative correlation was found between intra-lesional vessel density and normalized spectral power in interval III at lesion centres, suggesting that the higher the vessel density, the less vasomotion occurs.

As well as atypical and typical benign nevi, psoriasis was also chosen as a control lesion in our study, due to its similarity to SMM in terms of increased number and characteristics of microvessels^26^. Consistently with this similarity, we did not observe significant difference in average blood perfusion between SMMs and psoriasis lesions. On the contrary, SMMs showed a lower spectral power in the frequency intervals related to neurogenic and myogenic dependent vasomotion compared to psoriasis lesions, a finding which confirms the peculiarity of SMMs in terms of abnormal blood flow dynamics.

In addition to the diagnostic potential of these results, this method provides the opportunity for the characterization and ongoing monitoring of the tumour microvasculature, in terms of its dynamical properties. We have shown a reduction in spectral power of blood perfusion fluctuations in two frequency intervals in SMM, previously verified as being related to neurogenic and myogenic activity, i.e. those associated with vasomotion^20^,^21^,^22^,^23^. Two possible scenarios in which this behaviour could arise are 1) inefficient development of local blood vessels during angiogenesis, bypassing the usual connections to local regulatory mechanisms, or 2) changes in vessel reactivity due to alterations in the tumour microenvironment, or a combination of both. In both cases, the vascular network, and thus its functionality, will evolve with time as the tumour progresses. Using this method to quantify changes in physiological processes, from cardiac to endothelial function, as detected in melanoma blood perfusion, could be a very valuable tool during melanoma treatments, especially those based on targeting functional adaptations in tumour vasculature to increase treatment efficacy. It could also be useful as an additional functional parameter during investigations within the melanoma microenvironment, such as quantification of angiogenesis *in vivo*^27^, or during the determination of prognoses based on inflammation and interactions with endothelial cells^28^.

In conclusion, our study identified a microcirculatory diagnostic cut-off showing a very high accuracy in differentiating MMSs from atypical nevi. Whilst this cut-off clearly demonstrates the feasibility of this approach to the diagnosis of skin melanoma, further research is necessary before it can be recommended as a pre-excision biomarker for selecting skin melanocytic lesions that should be histologically examined. To translate these results to a larger scale, and facilitate the development of a specialized ‘melanometer’, the inherent heterogeneity of melanoma and atypical nevi lesions necessitates the recruitment of a larger cohort, as part of a multi-centre study. This should incorporate a wider range of lesion subtypes, for example Spitz nevi and melanoma *in situ*, to verify applicability of the results to all diagnostically difficult pathologies. Now that the parameters of the diagnostic test are established, a multi-channel LDF system can be developed for this specific purpose, and the knowledge that the lowest frequency interval of interest is 0.02 Hz will allow a reduction in measurement time to only 15 minutes. If successfully verified, this method is fast, inexpensive and non-subjective, and requires minimal training, providing huge potential for clinical use, and even accessibility to the public in the form of a specialized device for monitoring the evolution of skin lesions.

## Materials and Methods

### Subjects and plan of the study

In this cross-sectional study, subjects with a clinically and dermoscopically atypical nevus, suspected by an expert dermatologist to be a SMM, were recruited. A clinically atypical nevus was defined as a skin melanocytic lesion with one or more of the following clinical features: asymmetry, border irregularity, color variability and a diameter greater than 6 mm^8^. A dermoscopically atypical nevus was defined as a skin melanocytic lesion with dermoscopical features (pattern analysis) which may be indicative of SMM^8^. Further inclusion criteria were to be free from congestive heart failure, recent myocardial infarction, serious cardiac arrhythmia, chronic inflammatory diseases, neoplastic diseases, untreated arterial hypertension, severe liver diseases, untreated type 2 diabetes mellitus, type 1 diabetes mellitus, severe renal failure and hemodialysis treatment, in addition to being less than 81 years old. The exclusion criteria were introduced in order to create as homogeneous a group as possible for the purposes of blood flow monitoring, particularly excluding conditions which are also know to alter blood flow dynamics, such as cardiac conditions. This ensures that any differences observed may be reliably attributed to be a result of the presence of the lesion under study.

Healthy subjects with a clinically benign nevus and patients affected by non-active psoriasis were also recruited as control subjects, according to the same inclusion criteria (with the exception of being free from an inflammatory disease for psoriasis patients).

On the day subsequent to the recruitment, each subject underwent blood perfusion monitoring at the level of the lesion of interest, according to the protocol reported below. Following blood perfusion monitoring, subjects diagnosed with an atypical nevus underwent excision of the lesion, which was then histologically examined.

The protocol of this study was approved by the Ethical Committee of the University of Pisa (“Comitato per la sperimentazione clinica dei farmaci” of the Azienda Ospedaliero-Universitaria Pisana, Via Roma 67, 56126 Pisa, Italy), and was in accordance with the Helsinki Declaration as revised in 2000. All participants gave their written informed consent.

### Method of blood flow monitoring

Blood perfusion monitoring was carried out in the morning in a quiet room with air conditioning, whose temperature was systematically measured and ranged from 21 °C to 23.5 °C, whilst the subject was in supine position, using a single point LDF apparatus (Periflux PF4, Perimed, Järfälla, Sweden) equipped with an unheated probe (PF408). This allows skin blood flow to be detected in a tissue volume of around 1 mm and to be measured in perfusion units (PU) (1 PU = 10 mV). Subjects were asked to abstain from food, drugs, alcohol, coffee and tea for 3 hours prior to the LDF measurement and underwent this measurement after an acclimatization period of 20 minutes. The laser characteristics were: 780 nm wavelength, 10 Hz–19 kHz bandwidth, 0.1 s time constant, 32 Hz sampling frequency. Probe calibration was performed before each session, using a specialized device (Perimed, Järfälla, Sweden) and then affixed to the lesion of interest using a double sided adhesive disk. Blood flow signals were recorded continuously by an interfaced computer (Compaq, Hewlett Packard, Netherlands), equipped with software for data acquisition (Perisoft, Perimed, Järfälla, Sweden), for 30 minutes at the center of the lesion of interest and at the contralateral location on healthy skin, in the three groups of subjects. Immediately following these recordings, blood flow was monitored for 30 minutes at the margin of the lesion of interest in subjects with atypical and clinically typical nevi.

### Analysis of blood flow dynamics

Prior to analysis signals were inspected to identify movement artefacts or any other phenomena which were distinctly different from the inherent fluctuations. Five subjects were removed on the basis of this inspection, one with atypical nevus and four with typical benign nevi. These effects may arise from physiological or methodological factors, such as dry skin or the use of commercially available equipment which is not optimised for our purpose. We expect that further hardware development and optimization would eliminate these effects.

**Figure 4:**
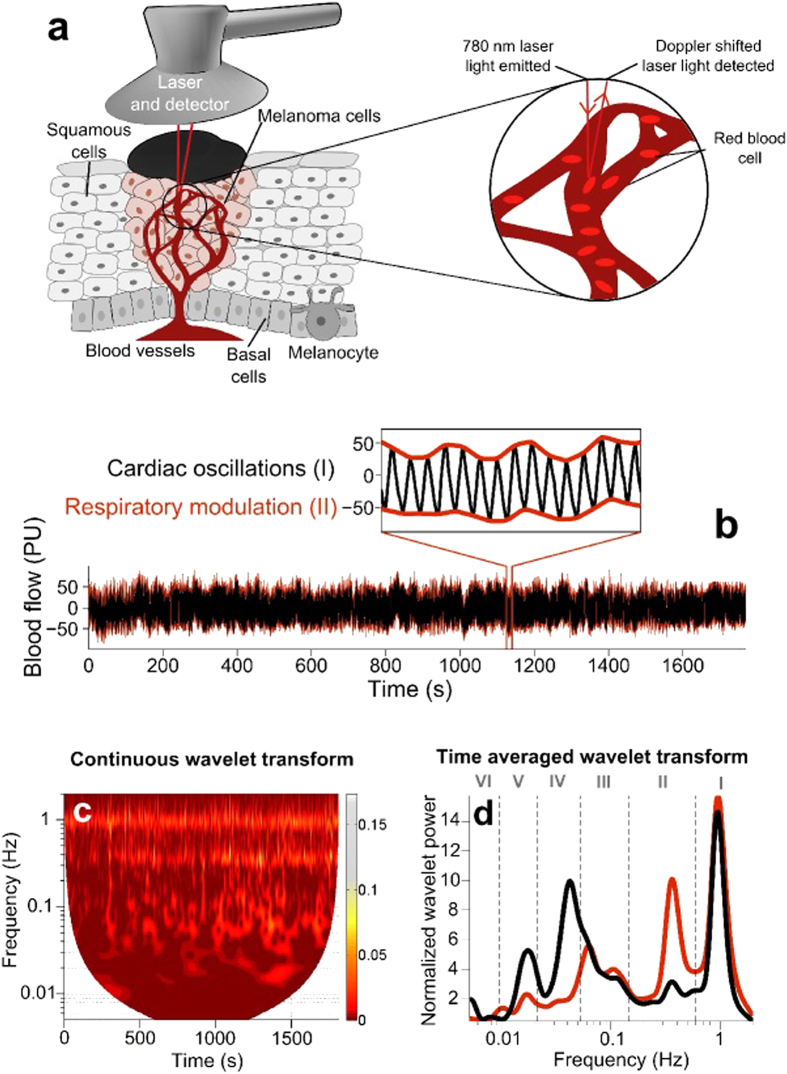
(**a**) A laser Doppler probe placed above a skin malignant melanoma. Skin malignant melanoma microvasculature and laser-Doppler effect are schematically shown. (**b**) Typical blood flow signals recorded from the center of a clinically benign skin nevus (black line) modulated by respiration (red line). (**c**) Continuous wavelet transform representation of the laser Doppler signal recorded from the center of a clinically benign skin nevus. (**d**) Time averaged wavelet transform representation of the laser Doppler signals recorded from the center of a clinically benign skin nevus (black line) and from the contralateral healthy skin site of the same subject (red line). This allows accurate visualization of the frequency content of the I-VI intervals defined in the Methods section.

Wavelet analysis was performed in the Department of Physics, Lancaster University, UK, using methods described in earlier works^20^,^22^. Time-frequency analysis methods, particularly the wavelet transform, have been shown to be necessary to fully characterize non-autonomous characteristics such as those which we know to be present in skin blood flow^18^. The continuous wavelet transform is given by


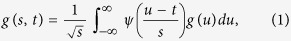


where *s* is a scaling factor, *t* is a location on the signal in time and *ψ* is the wavelet function, in this case the Morlet wavelet with a central frequency of 1. Time averaged wavelet spectral power was then calculated from the wavelet amplitude. Wavelet spectral power is analogous to the Fourier transform but more accurately represents time-variable dynamics and has much better resolution at lower frequencies as a result of its logarithmic frequency scale (Fig. 4(c,d)).

Wavelet spectral powers were divided into six frequency intervals for each LDF tracing: 0.61–2 Hz (interval I, related to heart activity), 0.145–0.6 Hz (interval II, breathing rate), 0.052–0.145 Hz (interval III, related to smooth muscle cell activity), 0.021–0.052 Hz (interval IV, related to neurogenic activity), 0.0095–0.021 Hz (interval V, related to nitric oxide-dependent endothelial activity) and 0.005–0.0095 Hz (interval VI, related to nitric oxide-independent endothelial activity)^20^,^21^,^22^,^23^. Due to the wide variation of location of the lesions, wavelet spectral powers were then normalized through division of all spectra by their total spectral power to allow direct comparison between recording sites and different groups, as it has been shown that recording location, reflecting various vascular densities, can result in different average blood flow values^29^. Note that the normalised values represent the *relative* contribution of each of the physiological processes that manifest within a certain frequency interval. As the normalized power contains information about oscillations in all frequency intervals of interest, and some increase and others decrease in amplitude, the change in the normalised power in each of the frequency intervals should be understood as being relative to the total changes.

### Histological examination

Following LDF monitoring, subjects with clinically atypical nevi underwent excision of the lesion of interest. Histological examination of the excised lesions was performed to determine the nature of the lesion and examine the surrounding vasculature. Intra and peri-lesional microvessels were highlighted with anti CD34 Mab (Ventana Medical System). Each sample was examined under low spectral power to identify the region with the highest number of microvessels (“hot spot”). Two (intra and peri-lesional) 250x fields (x 25 objective lens and 10x ocular lens) were evaluated to assess the number of microvessels (microvessel density).

### Statistical analysis

Data distribution was tested for normality by means of the Lilliefors test. Normal distributions were not consistently found in any data set, so all statistical tests used were non parametric. Group differences were investigated using the Kruskal Wallis ANOVA test. If significance was found, further differences between pairs of groups were tested using the Wilcoxon signed-rank test for paired data and the Wilcoxon rank-sum test for unpaired data. Linear regression was computed using the Theil-Sen estimator^30^, and correlation quantified by Kendall’s τ. Significance was set at *p *<* *0.05. To ensure adequate statistical power of the tests used, sample size and power calculations were performed with the pwr package using R. All significant differences were obtained with at least the generally accepted statistical power of 0.8, based on calculations of effect size (Cohen’s *d*) for each of the three test parameters (for complete calculations, see Supplementary Material).

## Additional Information

**How to cite this article**: Lancaster, G. *et al.* Dynamic markers based on blood perfusion fluctuations for selecting skin melanocytic lesions for biopsy. *Sci. Rep.* **5**, 12825; doi: 10.1038/srep12825 (2015).

## Supplementary Material

Supplementary Information

## Figures and Tables

**Table 1:** Histological characteristics of all examined lesions.

Lesion type	Number of patients	Total subjects (male)	Age range (median)	Locations
Malignant melanoma				
Superficial spreading	6	10 (4)	39–80 (62)	Abdomen (1), leg (3), flank (1), shoulder (1), arm (2), face (2)
Nodular	3			
Lentigo	1			
Atypical/dysplastic nevi				
Compound	24	33 (18)	17–73 (41)	Clavicle (1), leg (6), chest (1), flank (1), shoulder (6), lumbar (2), thorax (7), gluteal (2), abdomen (4), arm (2), foot (1)
Junctional	6			
Basocellular epithelioma	1			
Dysplastic	2			
Benign nevi				
Compound	3	37 (18)	21–78 (46)	Arm (3), ankle (1), shoulder (4), foot (3), leg (6), flank (1), thorax (2), front (1), side (1), abdomen (8), chest (4), breast (2), back (1)
Junctional	4			
Dermal	2			
Blue	1			
Acral compound	1			
Clinically benign	26			
Psoriasis				
Clinically diagnosed	9	9 (8)	35–76 (63)	Leg (6), shoulder (1), arm (1), flank (1)

**Table 2:** Histological characteristics of malignant melanoma lesions.

Sex	Age (years)	Tumor size (cm)	Clark level	Breslow depth (mm)	Ulceration
M	58	1.2 × 0.7 × 0.4	III-IV	3.0	yes
F	43	0.5 × 0.4	III	0.4	no
F	39	0.4 × 0.3	II	0.2	no
F	80	1.8 × 1.3	IV	0.8	no
F	51	1.1 × 0.7	II	0.45	no
M	74	1.5 × 0.9	II	0.45	no
M	58	1.7 × 1.0	II	0.35	no
F	80	1.1 × 0.7 × 0.3	IV	3.1	no
F	80	2.5 × 2.5 × 1.5	IV	15.0	yes
M	64	0.6 × 0.5	III	0.7	no

Anonymized subject data is provided in the supplementary data set which can be found here http://www.lancaster.ac.uk/library/rdm/data-catalogue/researchdata-13/

**Table 3:** Histological characteristics of atypical nevi.

Characteristics of atypical nevi			
	Slight	Moderate	Severe
Compound	7	17	7
Junctional	3	6	1
Dermal	0	2	1
Total	10	25	9

**Table 4:** 

(a) Recording site	Malignant melanoma (10)	Atypical nevus (33)	Typical benign nevus (37)	Psoriasis (9)	*p* (Kruskal Wallis)
Center	126.8 (80.0–158.6)	15.2 (10.1–29.1)	18.6 (9.5–23.9)	111.3 (85.8–125.6)	0.0000
Margin	77.1 (53.4–91.8)	16.6 (10.4–32.5)	19.1 (12.2–28.0)		0.0004
Contralateral	14.2 (11.8–20.6)	12.5 (10.8–16.0)	13.9 (11.7–16.9)	15.7 (9.8–17.5)	0.5662
*p* (Sign rank)	0.0020	0.0179	0.3940	0.0039	
(b) FI - I					
Center	10.8 (8.2–14.3)	4.9 (2.2–7.3)	4.4 (2.4–5.5)	6.6 (3.8–8.3)	0.0003
Margin	9.4 (5.8–12.2)	3.6 (2.4–6.7)	3.5 (1.9–5.4)		0.0034
Contralateral	5.2 (2.7–7.1)	2.8 (1.2–4.8)	3.8 (2.6–5.7)	2.3 (1.6–4.7)	0.1331
*p* (Sign rank)	0.0020	0.0004	0.7229	0.0078	
FI - II					
Center	1.0 (0.8–1.8)	1.8 (1.3–3.0)	2.2 (1.3–3.0)	1.3 (0.7–1.8)	0.1571
Margin	1.6 (0.9–2.3)	2.1 (1.4–3.5)	2.3 (1.3–3.7)		0.5163
Contralateral	1.5 (1.2–2.8)	1.9 (1.5–2.8)	2.0 (1.2–3.1)	2.8 (2.2–4.4)	0.6101
*p* (Sign rank)	1	0.6877	0.3305	0.0039	
FI - III					
Center	1.7 (0.9–2.7)	4.7 (2.6–7.5)	4.8 (3.6–7.6)	3.0 (2.8–10.3)	0.0006
Margin	2.8 (2.0–4.0)	4.8 (3.0–8.0)	5.3 (3.6–8.2)		0.0230
Contralateral	5.7 (2.1–6.7)	4.7 (3.2–7.0)	4.6 (3.0–6.0)	4.0 (2.2–5.0)	0.7257
*p* (Sign rank)	0.0020	0.9501	0.1184	0.2500	
FI - IV					
Center	1.0 (0.6–1.7)	1.9 (1.4–3.5)	2.6 (1.9–4.3)	1.8 (1.6–3.0)	0.0005
Margin	1.2 (1.0–2.0)	1.8 (1.3–3.1)	2.4 (1.5–4.5)		0.0408
Contralateral	3.1 (1.8–5.1)	3.9 (3.3–5.7)	4.2 (2.5–5.4)	3.3 (2.6–4.8)	0.4845
*p* (Sign rank)	0.0020	0.0001	0.0074	0.0977	
FI - V					
Center	1.0 (0.6–1.7)	1.8 (1.0–3.7)	2.0 (1.5–2.6)	1.5 (1.2–1.8)	0.0746
Margin	1.2 (0.7–2.5)	1.6 (0.9–2.3)	1.8 (1.0–3.4)		0.6096
Contralateral	2.2 (1.4–2.5)	3.4 (1.5–4.4)	3.0 (1.2–3.7)	3.2 (2.1–3.5)	0.3417
*p* (Sign rank)	0.0039	0.0049	0.1868	0.0547	
FI - VI					
Center	1.9 (0.6–3.0)	1.5 (0.8–2.4)	1.5 (1.1–2.7)	2.6 (1.4–4.9)	0.8659
Margin	1.2 (0.6–1.9)	1.5 (0.9–2.4)	1.6 (1.0–2.6)		0.5098
Contralateral	0.8 (0.7–1.4)	1.6 (1.1–3.4)	2.1 (0.9–3.9)	2.8 (1.6–5.2)	0.1867
*p* (Sign rank)	0.6953	0.4915	0.7229	0.9102	

(**a**) Median and inter quartile ranges of the mean blood flow values, expressed in perfusion units, detected at the level of the examined lesions and contralateral skin sites. (**b**) Median and inter quartile ranges of normalized power values for the six individual frequency intervals investigated at the level of the examined lesions and contralateral skin sites. Sign rank *p* values calculated between lesion centers and contralateral skin. Kruskal Wallis values calculated between melanoma, atypical nevi and typical benign nevi. FI = frequency interval.
