# Erratum: Dynamic markers based on blood perfusion fluctuations for selecting skin melanocytic lesions for biopsy

**DOI:** 10.1038/srep20162

**Published:** 2016-02-09

**Authors:** Gemma Lancaster, Aneta Stefanovska, Margherita Pesce, Gian Marco Vezzoni, Barbara Loggini, Raffaele Pingitore, Fabrizio Ghiara, Paolo Barachini, Gregorio Cervadoro, Marco Romanelli, Marco Rossi

Scientific Reports 5: Article number: 1282510.1038/srep12825; published online: 08112015; updated: 02092016

In the HTML version of this Article, Gemma Lancaster and Aneta Stefanovska were incorrectly listed as being affiliated with ‘Department of Clinical and Experimental Medicine, University of Pisa, Italy’. The correct affiliation is listed below:

Department of Physics, Lancaster University, UK.

In addition, Margherita Pesce, Gian Marco Vezzoni, Paolo Barachini, Gregorio Cervadoro, Marco Romanelli and Marco Rossi were incorrectly listed as being affiliated with ‘Department of Physics, Lancaster University, UK.’ The correct affiliation is listed below:

Department of Clinical and Experimental Medicine, University of Pisa, Italy

These errors have now been corrected.

An incorrect Supplementary Information file was inadvertently published with this Article. The correct Supplementary Information now accompanies the Article.

