# Supplementary material for: Dynamic markers based on blood perfusion fluctuations for selecting skin melanocytic lesions for biopsy
Source: Sci Rep. 2015 Aug 11;5:12825. doi: 10.1038/srep12825 (PMC4542467; doi:10.1038/srep12825)
Supplement: Supplementary Information [file srep12825-s1.pdf]

Dynamic markers based on blood perfusion fluctuations for selecting skin melanocytic lesions for biopsy - Supplementary material

M. Rossi[1], G. Lancaster[2], M. Pesce[1], G. M. Vezzoni[1], B. Loggini[3], R. Pingitore[3], F. Ghiara[1], P. Barachini[1], G. Cervadoro[1], M. Romanelli[1] and A. Stefanovska[2]

[1] *Department of Clinical and Experimental Medicine, Pisa University, Italy*
[2] *Department of Physics, Lancaster University, UK*
[3] *Department of Translational Research and New Technologies in Medicine, Pisa University, Italy*

Author contributions

A. Stefanovska conceived and designed the study. M. Rossi planned and managed the study. M. Pesce performed the LDF experiments. G. M. Vezzoni, F. Ghiara, P. Barachini, G. Cervadoro and M. Romanelli were involved in recruitment and diagnosis. B. Loggini and F. Pingitore performed histological examinations. G. Lancaster performed the data analysis and with M. Rossi drafted the manuscript. All authors edited and approved the manuscript.

Introduction to time-frequency analysis

Example 1: Consider 2 simple sine waves of frequency 1 Hz and 0.1 Hz, their sum and their FFT:

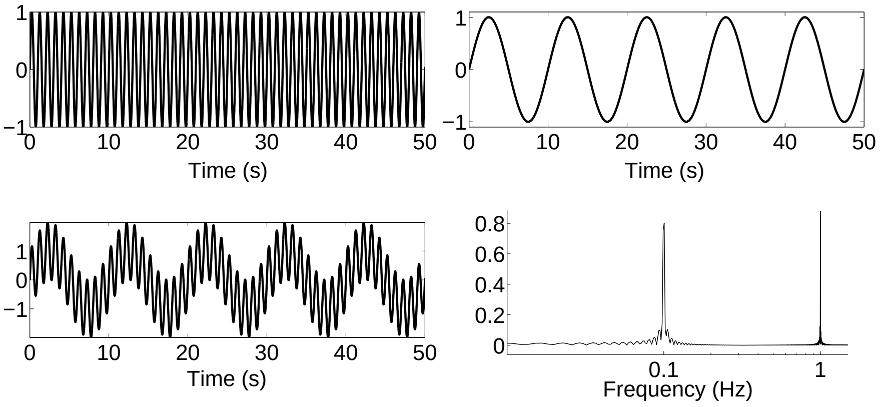

Figure 1: Top left - 1 Hz sine wave, Top right - 0.1 Hz sine wave, Bottom left - addition of sine waves, Bottom right - Fourier transform of composite signal.

Computationally, the Fourier transform can be implemented using fast Fourier transform (FFT) algorithms (actually the discrete Fourier transform (DFT)). The FFT of the composite sine wave can be seen in Figure 1. Whilst this is ideal for signals like those above, which are *stationary*, i.e. their frequencies do not vary with time, the Fourier transform can misrepresent non-stationary signals.

The continuous wavelet transform

Example 2: A chirp signal is a signal in which the frequency can increase or decrease with time.

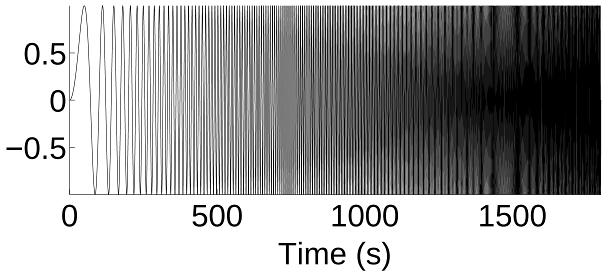

Figure 2: Quadratic chirp signal, sin(2π*0.0001*time^2)

The Fourier transform cannot fully describe this signal, as all time information is lost. The short time Fourier transform (STFT) has been employed in attempts to solve this problem, which divides the signal into windows in which the signal can be approximated to a stationary one. Here, the window size must be carefully chosen depending on whether the user requires good time resolution or good frequency resolution; one cannot have both simultaneously. The continuous wavelet transform allows good resolution in both domains.

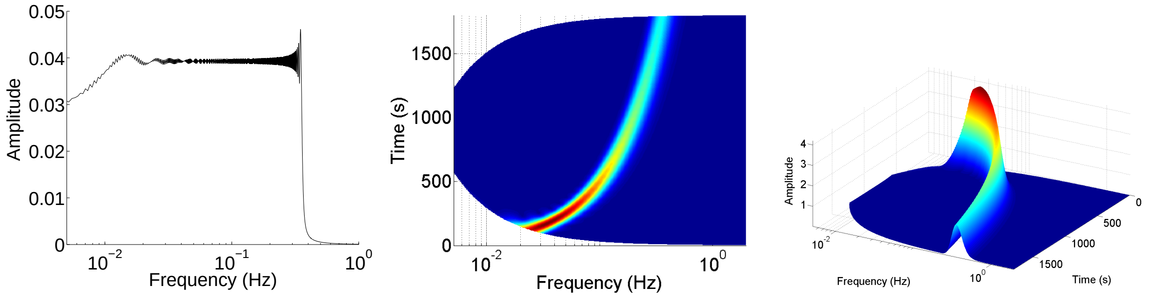

Figure 3: Left - FFT of chirp signal in Figure 2, centre & right- continuous wavelet transform of chirp signal.

The continuous wavelet transform is given by

$$g(s,t) = \frac{1}{\sqrt{s}} \int_{-\infty}^{\infty} \psi \left(\frac{u-t}{s} \right) g(u) du$$

where s is a scaling factor, t is a location on the signal in time and ψ is the wavelet function [3]. The wavelet transform is obtained by moving a wavelet function along all locations of the signal. For each location, a full range of scales of the wavelet are used, and can be adjusted depending on the range of frequencies that is expected. The value obtained from the convolution of the wavelet function with the signal at these times and frequencies will be large if there is a good match (if the signals are out of phase, a large value will still be given but it will be negative). In this way, a whole picture of a signal can be created, with the axes time, frequency and amplitude, which is the value of the transform at each scale.

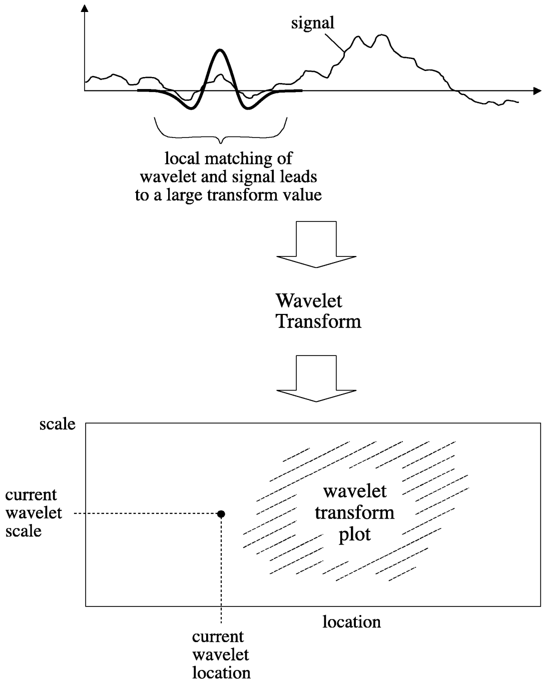

Figure 4: Example of the translation of the wavelet across a signal in time, showing an area of local matching (top). This wavelet is then calculated for different scales and the process repeated. The results of the convolution of the wavelet with the time series are plotted at each point (bottom). Figure from [2].

There are many different wavelets, each having their own applications. In this analysis, we use a complex wavelet. With complex wavelets, the phase and amplitude components of a signal can be separated. The wavelet used was the Morlet wavelet, a complex wave within a Gaussian envelope, which has unit standard deviation. The real and imaginary sinusoids differ in phase by a quarter of a period. The Morlet wavelet is defined as

$$\psi(u) = \frac{1}{\sqrt[4]{\pi}} e^{i2\pi f_0 u} e^{-u^2/2}$$

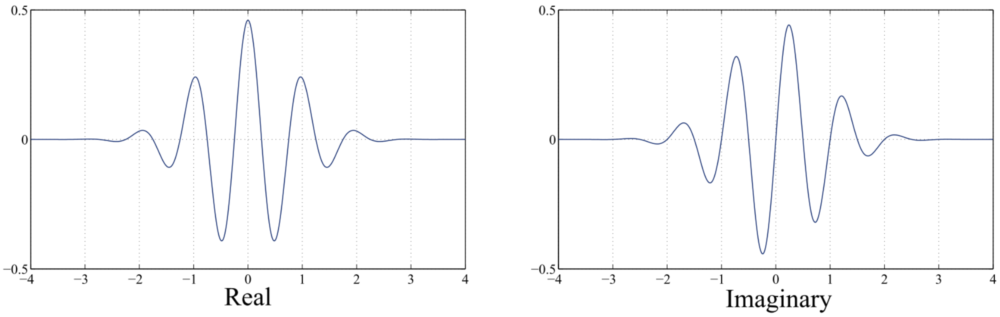

Figure 5: The real and imaginary parts of the complex Morlet wavelet.

Implementation

An example algorithm for the calculation of the CWT is below:

1) Decide the frequency limits within which you require the CWT, and create a logarithmic scale array, noting that with the Morlet wavelet of f_0 1 scale=1/frequency.

2) Calculate the wavelet for each scale and pad with zeros to create an array, W, the same length as the original time series, T.

3) The convolution required in the calculation of the CWT can be implemented using the convolution theorem:

$$g * h \equiv G(f)H(f)$$

i.e. the Fourier transform of the convolution is the product of the individual Fourier transforms of the wavelet and the time series [1]. So, the convolution of the time series and the wavelet is equal to the inverse Fourier transform of the product of their individual Fourier transforms.

Codes to implement the continuous wavelet transform, as well as video instructions, can be found at http://www.physics.lancs.ac.uk/research/nbmphysics/diats/tfr/

Application to blood flow signals

It has been shown previously that there are at least six distinct oscillations present in skin blood perfusion signals [4, 5](see Table 1). It has also been demonstrated that these oscillations are non-stationary, as are many signals of biological origin. Whilst many previous studies have characterised blood flow signals in terms of their average values, this approach discards a lot of valuable information. Three blood perfusion signals recorded in the current study from a subject with an atypical nevus are shown in Figure 6. It is immediately apparent that there are oscillations present, and so we seek to extract frequency information. Applying the Fourier transform to the centrally recorded signal reveals a peak in the cardiac frequency interval as expected. The Fourier transform has a linear frequency scale, and it is apparent from Figure 7(a) that it would be difficult to separate any distinct oscillations present below 0.2 Hz. Showing the same Fourier transform on a logarithmic frequency scale (Figure 7(b)) shows the reduction in frequency resolution at lower frequencies. To overcome this problem, and also the requirement of the Fourier transform for stationarity of signals, the continuous wavelet transform was employed, which shows time and frequency information simultaneously, allowing more accurate representation of non-autonomous dynamics [6]. To be able to compare signals, we can calculate the time average of this wavelet transform, which will then provide us with a frequency spectrum which is analogous to the Fourier transform, but provides much better frequency resolution in the frequency intervals in which we are interested. This approach also provides the opportunity to extract instantaneous frequencies of specific oscillations.

FI	Oscillation frequency (Hz)	Physiological process
I	0.6 - 2	Cardiac activity (heart rate)
II	0.145 - 0.6	Respiration (breathing rate)
III	0.052 - 0.145	Myogenic activity (contraction/relaxation of vessels)
IV	0.021 - 0.052	Neurogenic activity (innervation of vessels)
V	0.0095 - 0.021	Nitric oxide (NO) related endothelial activity
VI	0.005 - 0.0095	NO independent endothelial activity

Table 1: Distinct frequency intervals found in skin blood perfusion, their frequencies and the physiological process to which they have been attributed. FI = frequency interval.

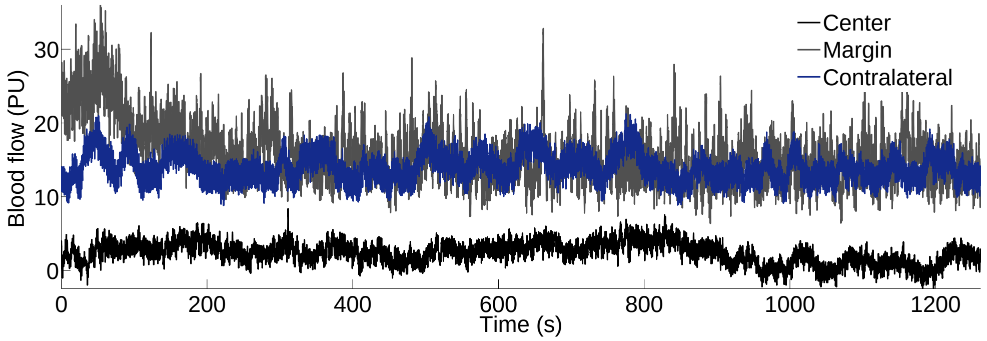

Figure 6: Blood perfusion signals recorded in an atypical nevus, at the centre (black), at the margin (gray), and on contralateral healthy skin (blue).

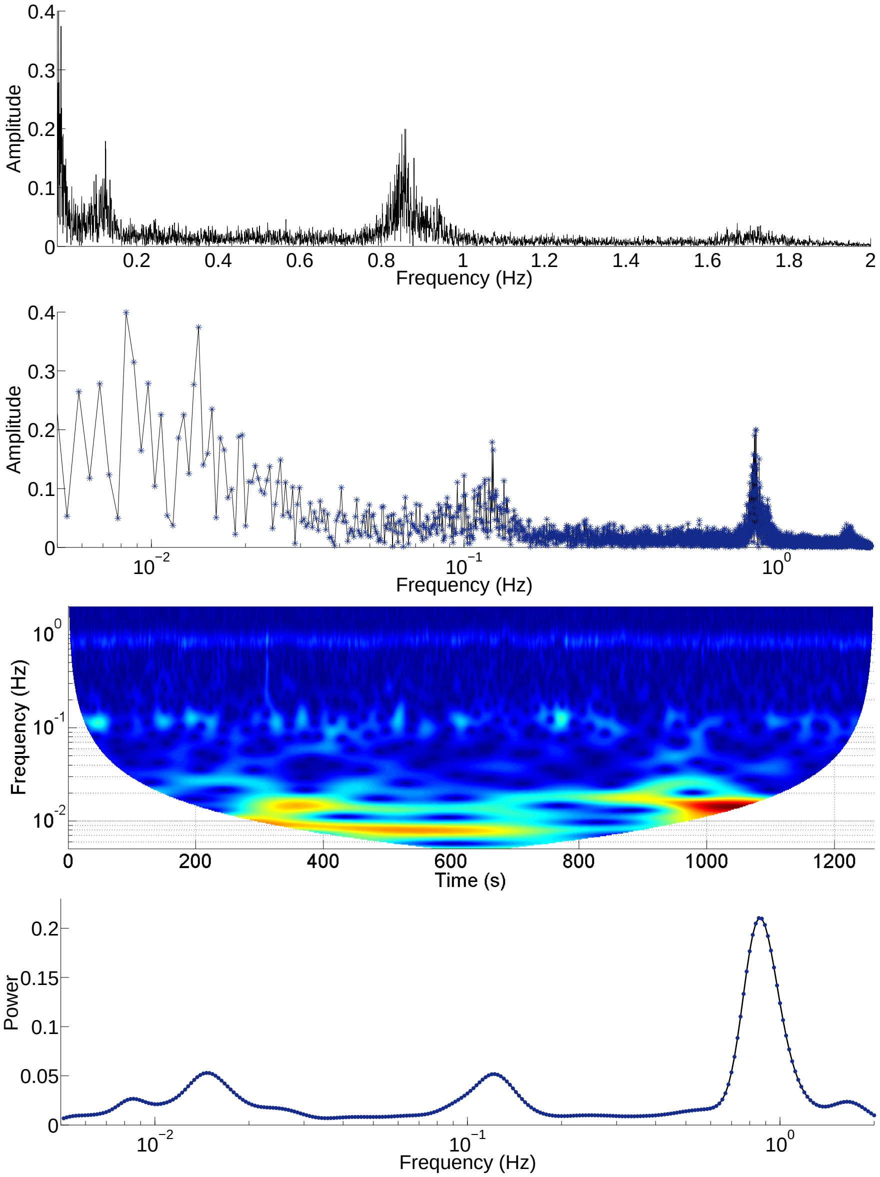

Figure 7: a) FFT of the blood flow signal recorded in the center of AN6 plotted on a linear frequency scale, b) The same FFT in a) plotted on a log scale, note the reduction in resolution at lower frequencies, the blue markers demonstrate the frequencies at which the FFT was calculated, c) the continuous wavelet transform of the same signal, allowing the evolution of the frequencies of each component to be followed in time, d) the time average of the wavelet transform shown in c) which provides much better frequency resolution at lower frequencies such as those which we are investigating.

Analysis method

A step by step explanation of the analysis is below:

1) Mean values were calculated of all blood perfusion signals and compared between and within groups using the appropriate statistical tests.

2) All signals were detrended using a moving average method, using a window size of 200 seconds, in order to remove trends and frequency information below the lowest frequency interval to be investigated (0.005 Hz). The mean was also subtracted from the signals.

3) Continuous wavelet transforms were calculated for all detrended blood perfusion signals, in the frequency interval 0.005 Hz to 2 Hz. The central frequency of the Morlet wavelet (f_0) was 1.

4) Time averaged wavelet powers were calculated for each signal through calculation of the mean of all time domain information at each frequency.

5) Due to the widely varying locations of lesions, the time averaged wavelet power spectra were normalized by dividing the power at each frequency by the total power present in the spectrum.

6) These normalized power spectra were compared within and between groups, at each individual frequency (the frequency domain was split into 277 points)(see Figure 8).

7) To allow comparison between different frequency intervals in normalized powers, total power in each interval was calculated. Because the number of points in each interval was not equal, due to different interval lengths, this value was then divided by the number of points in each interval (see Figure 9).

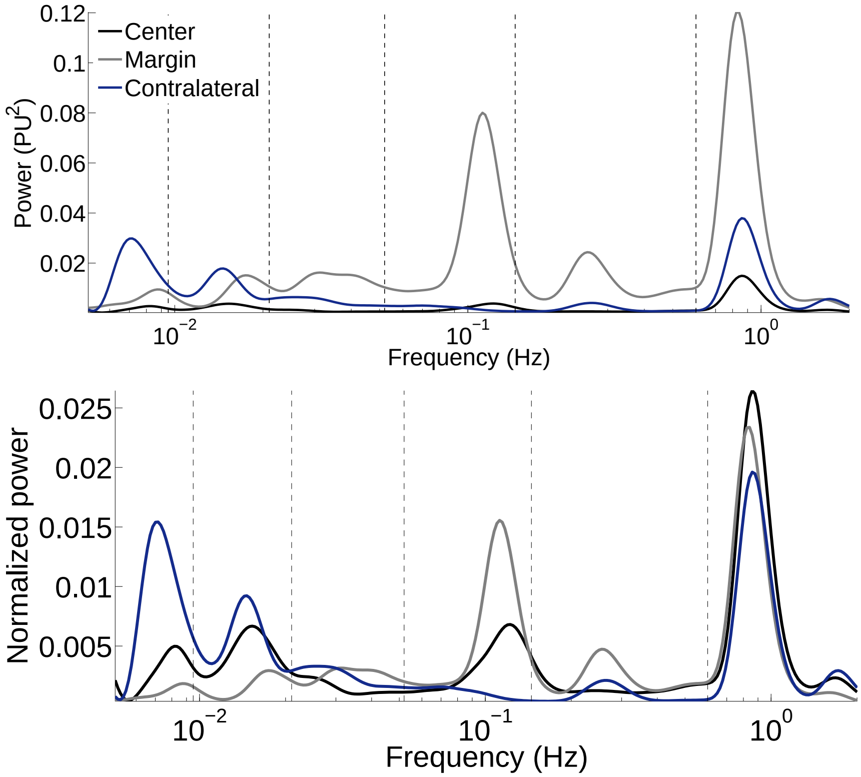

Figure 8: Absolute (top) and normalized (bottom) time averaged wavelet power calculated from the LDF signals in Figure 6.

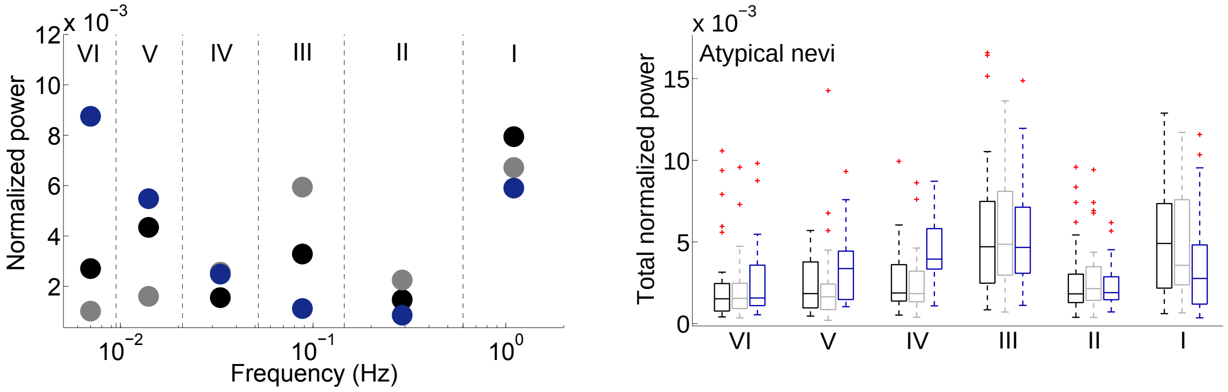

Figure 9: Left - example of total power values calculated for the six frequency intervals from normalized powers. Each circle represents a total power value, where black is from the center of the lesion, gray is from the margin and blue is from contralateral skin. These values can then be compared between groups. All results from atypical nevi (n=33) are shown in the figure on the right.

Supplemental figures

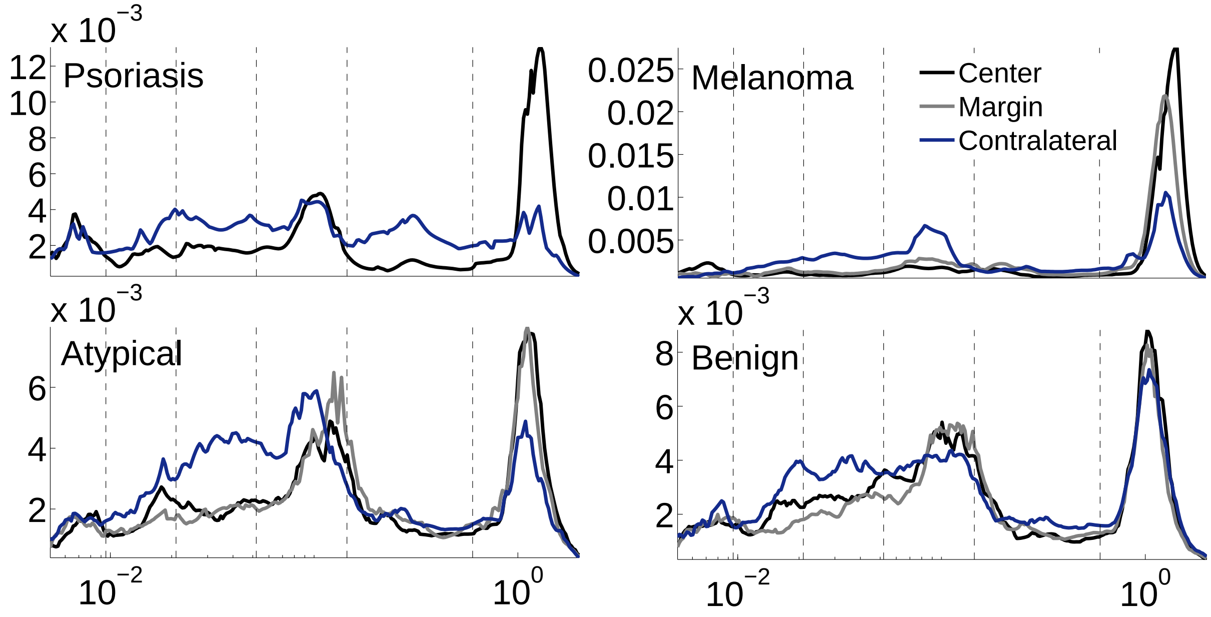

Figure 10: Normalized time averaged wavelet transforms for each group. Psoriasis, n=9, melanoma, n=10, atypical, n=33, benign, n=37.

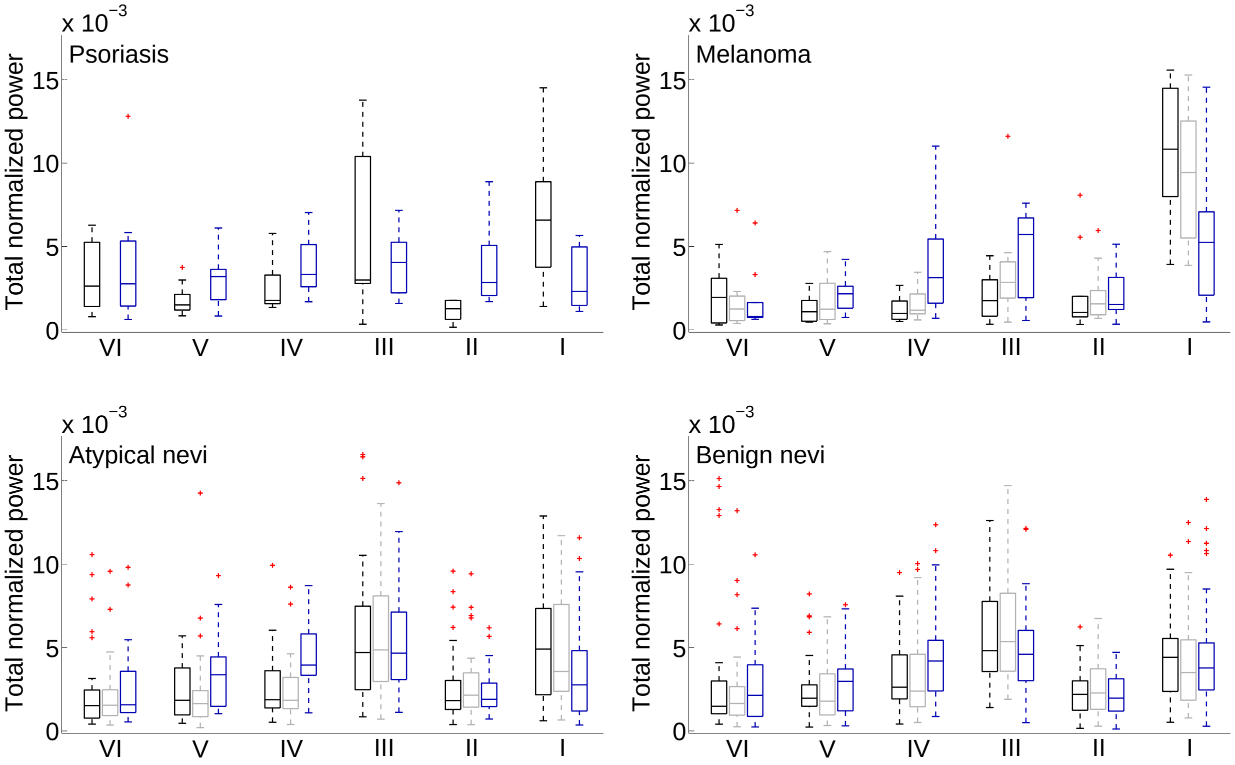

Figure 11: Boxplots of total normalized power in all groups, comparing signals from lesion centers (black), to margins (gray) and contralateral skin (blue). P values from the Wilcoxon sign rank test between central and contralateral signals are shown in the table below.

Frequency interval	Psoriasis	Melanoma	Atypical	Benign
I	0.0078	0.0020	0.0004	0.7229
II	0.0039	1	0.6877	0.3305
III	0.25	0.0020	0.9501	0.1184
IV	0.0977	0.0020	0.0000	0.0074
V	0.0547	0.0039	0.0049	0.1868
VI	0.9102	0.6953	0.4915	0.7229

Table 2: Wilcoxon sign rank test P values calculated between total normalized powers at centers vs. contralateral for all groups.

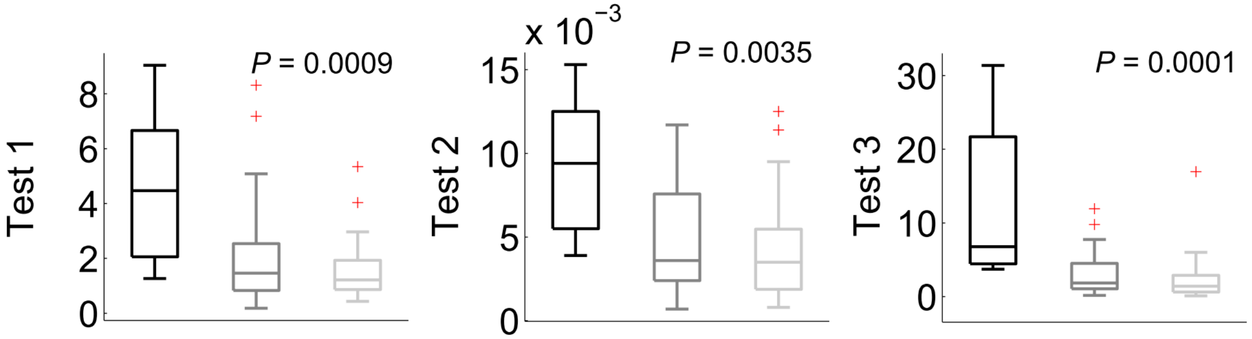

Figure 12: Boxplots showing the differences between groups (melanoma - black, atypical nevi - dark gray, benign nevi - light gray) in the three variables which make up the diagnostic test, with their corresponding P values, as reported in the main text. Test 1 is calculated from the mean blood flow at the lesion margin divided by the mean blood flow at the contralateral location. Test 2 is based on the normalized cardiac powers in lesion margins. Test 3 is the total power in the cardiac interval (interval I) divided by the total power in the neurogenic interval (interval IV).

Subject	Melanoma				Atypical				Clinically atypical				Benign			
	Variable 1	Variable 2	Variable 3	Score	Variable 1	Variable 2	Variable 3	Score	Variable 1	Variable 2	Variable 3	Score	Variable 1	Variable 2	Variable 3	Score
**1**	1.2643	0.0041	4.4356	0	5.0837	0.0108	9.7875	0	1.2417	0.0125	4.2689	1				
**2**	6.2254	0.0089	21.684	0	0.92536	0.0025	0.80087	3	2.3998	0.0012	0.33911	2	0.9909	0.0035	4.6652	2
**3**	6.6621	0.0039	6.669	0	2.5767	0.0029	7.7403	1	0.71437	0.0034	4.2647	2	0.6389	0.0057	3.2909	2
**4**	3.6643	0.0099	4.6145	0	0.81628	0.0015	0.17741	3	0.93964	0.0017	0.14775	3	1.0104	0.0052	2.3501	2
**5**	6.7023	0.0067	4.3726	0	2.2365	0.0102	1.2338	1	0.99958	0.0019	1.5214	3	1.8712	0.008	1.4072	1
**6**	4.901	0.0055	20.07	0	1.0892	0.0067	5.1212	1	1.7329	0.0009	0.57583	2	1.1293	0.0012	1.0817	3
**7**	9.0407	0.0112	3.7076	0	1.5986	0.0007	1.0994	2	0.85265	0.0049	1.9438	2	1.2395	0.0058	0.64375	2
**8**	1.8805	0.0125	28.931	0	0.8335	0.0012	0.6167	3	4.0325	0.0014	1.3788	2	0.77142	0.0008	0.12037	3
**9**	2.0576	0.0153	31.357	0	0.73054	0.0063	1.4063	2	1.2141	0.0018	2.8379	3	2.5542	0.0034	1.3377	2
**10**	4.0369	0.0133	6.854	0	8.3143	0.0104	0.31753	1	0.90943	0.0054	3.7674	1				
**11**					0.98156	0.0117	5.4646	1	1.6451	0.0054	16.941	0	1.6902	0.0044	2.3916	1
**12**					1.0541	0.003	1.8687	3								
**13**					0.73152	0.0007	0.2151	3					0.59265	0.0012	0.23481	3
**14**					0.28811	0.0058	2.6311	2					2.228	0.0036	3.0377	2
**15**					7.183	0.0065	2.912	1					0.83804	0.0029	1.7187	3
**16**					2.3337	0.0024	1.8559	2					2.2744	0.0042	1.4581	1
**17**					3.8104	0.003	4.311	1					1.6672	0.0028	1.2079	2
**18**					0.36427	0.0035	0.91603	3					2.97	0.0022	1.1839	2
**19**					1.0816	0.0036	5.3285	2					0.8674	0.0031	0.68864	3
**20**					0.61746	0.001	0.15699	3								
**21**					2.5235	0.0028	6.3356	1					1.9455	0.0065	0.85286	1
**22**					3.5254	0.0038	7.1479	0					5.3451	0.0095	6.0078	0
**23**					0.96907	0.0033	2.822	3					0.68712	0.0011	0.11262	3
**24**					3.6705	0.0112	11.93	0					0.88737	0.002	0.31324	3
**25**					0.64243	0.0017	1.2743	3					1.8671	0.0081	0.41483	1
**26**					1.9413	0.0109	1.6392	1					0.77943	0.0042	0.36884	2
**27**					1.4581	0.0057	1.8287	1					1.9361	0.0043	0.71955	1
**28**					1.571	0.0024	3.321	2					1.9236	0.0114	4.861	0
**29**					2.8634	0.0104	1.677	1					0.98053	0.0073	2.1113	2
**30**					0.18158	0.0036	3.3037	3					0.43292	0.0034	2.2374	3
**31**					2.1634	0.0063	1.3712	1								
**32**					0.93941	0.0108	3.1974	2								
**33**					1.9624	0.0018	0.95016	2								
**Below cut off**	0	0	0		16	18	24	**30**	7	7	7	**10**	14	13	23	**24**
**Sensitivity**	100.00%	100.00%	100.00%	**100.00%**												
**Specificity**					48.48%	54.55%	72.73%	**90.91%**	63.64%	63.64%	63.64%	**90.91%**	53.85%	50.00%	88.46%	**92.31%**

Table 3: Table of all calculated values involved in the diagnostic test, for each group and their corresponding scores. A score of 0 gives a result of melanoma, a score above 0 gives a result of non-melanoma. Sensitivities and specificities for each individual variable, and their combined values are given.

Calculation of sample size

Sample size is rarely reported in diagnostic accuracy studies [8], so no standard treatment of the problem exists. Consider our main hypothesis, that: *statistically significant alterations in skin blood flow dynamics will be observable when comparing skin malignant melanoma with atypical naevi and healthy skin.* Also consider our secondary aim, to consider the utilisation of any observed differences in the noninvasive diagnosis of skin melanoma.

Based on the aims and outcomes of our study, we may conclude that our study is an observational study. Important considerations in sample size calculation are as follows [9]

- The null hypothesis - there is no difference between blood flow dynamics in melanoma and atypical naevi.
- The alternative hypothesis - there is a difference between blood flow dynamics in melanoma and atypical naevi.
- The level at which we wish to avoid a type I error (pα) - A type I error is the incorrect rejection of the null hypothesis, i.e. finding differences where there are none, usually set to 0.05.
- The level at which we wish to avoid a type II error (pβ) - A type II error in the incorrect acceptance of the null hypothesis, i.e. stating there is no difference when there is, typically 0.8.
- What difference do we want to detect? What is the magnitude of this difference?
- Standard deviations of the variables of interest.
- The distribution of the data. Many statistical tests and sample size calculations are based on assumptions of normal distributions. In our study, the non-normal distribution of data warranted the use of non-parametric tests. The same criteria which caused us to use these tests will apply to sample size calculations, and inevitably lead to the need for larger sample sizes.

Power of significance tests

Statistically significant differences in blood flow were revealed between groups. Here we assess the statistical power of the tests used to obtain these results. As not all distributions of final test parameters were normal (as determined by the Lilliefors test), these differences were found using non parametric tests, namely the Wilcoxon rank sum test for unpaired data (the parametric equivalent is the t test for independent samples), and the signed rank test for paired data (parametric equivalent - paired t test). When comparing more than one group, the Kruskal Wallis ANOVA test was used (parametric equivalent - one way analysis of variance, F test). The statistical power of these tests will depend on sample size.

Sample size, effect size, significance level and power are related, and given any three, we can simply calculate the fourth, based on a normal distribution. When we cannot assume a normal distribution, the situation is more complex. Nevertheless, we begin by calculating the required parameters from the existing data, in order to eventually give an estimate of the required sample size, our missing variable. Asymptotic relative efficiency (ARE),

loosely describes the ratio of sample sizes required (parametric to non-parametric) for a parametric procedure to have the same ability to reject a null hypothesis as the corresponding nonparametric procedure. It has been shown that the ARE of the Wilcoxon-Mann-Whitney (rank sum) test is always at least 0.864, regardless of the underlying population [7]. Although this value may increase depending on the data, we will use this worst case scenario in the following calculations, and therefore increase all calculated sample sizes by 15%.

Effect size

We have three sets of parameters to consider which were used in the final test. Effect sizes are calculated for all cases, and the smallest effect size will be used in calculations, as this will result in the largest requirement for sample size. In these comparisons, only the difference between parameters in the melanoma and atypical naevi groups are considered, as atypical naevi provide the biggest diagnostic challenge, and from observations are more similar to melanoma in their blood flow characteristics than benign naevi, thus providing a more rigorous test of statistical power.

Test 1 - Ratio of mean blood perfusion at lesion margins and contralateral skin, recorded simultaneously.
Test 2 - Normalized spectral power of cardiac interval at the lesion margin.
Test 3 - Ratio of total spectral power in the cardiac and neurogenic frequency intervals in blood perfusion at lesion centres.

Effect size can be calculated as Cohen's d,

$$d = \frac{\bar{x}_1 - \bar{x}_2}{s},$$

where s is the standard deviation and $\bar{x}_1 - \bar{x}_2$ is the difference between the two means of the samples. The standard deviation required can be a pooled standard deviation of the two groups, but this should not be used if the two standard deviations are likely to be systematically different. Therefore, we use the control standard deviation in the calculation. We therefore obtain effect sizes for tests 1, 2 and 3 of 1.393, 1.095 and 3.536, respectively. We take the effect size of parameter 2, the smallest effect size, forward into the sample size calculation. Sample size can be calculated using the pwr package in R.

We use R to compute the required sample size of the melanoma group (**n1**), to perform a two sample, independent, one tailed, t-test, based on the following input [10]:

- **n2** - sample size of the atypical naevi group. We currently have 33 subjects, but because the tests used are non parametric, this will be reduced according to the extra 15% requirement explained above. The effective sample size thus becomes 28.696 subjects. Rounding down gives n2=28.
- **d** - effect size - we use the effect size calculated from parameter 2 - 1.095
- **sig.level** - probability of a type I error, α. As standard, we set this to 0.05
- **power** - the probability that the test will correctly reject the null hypothesis (1-β, where β is the probability of a type II error). As standard, we set this to 0.8.

- **alternative** - specifies whether the test is two tailed, or one tailed (less or greater). Here we use greater.

```
pwr.t2n.test(n2=28, power=0.8, d=1.095, sig.level=.05, alternative="greater")

     t test power calculation

             n1 = 6.658445
             n2 = 28
              d = 1.095
      sig.level = 0.05
          power = 0.8
    alternative = greater
```

The test indicates that we require a sample size of at least 6.658 (which we round up to 7) to provide adequate statistical power. Including a further 15% to account for the non-parametric nature of our tests leads to a minimum required sample size of 8.05, which we round up to 9. As we currently have 10 subjects in the malignant melanoma group, we can reject the null hypothesis that there are no differences in blood flow dynamics in the cardiac interval. As all other test parameters resulted in even larger effect sizes, we would expect to also be able to reject the null hypotheses that those parameters do not differ between groups. We can demonstrate this by using the effect size from parameter 1 and observing the reduction in n1 that is required to maintain sufficient statistical power:

```
pwr.t2n.test(n2=28, power=0.8, d=1.393, sig.level=.05, alternative="greater")

     t test power calculation

             n1 = 3.789535
             n2 = 28
              d = 1.393
      sig.level = 0.05
          power = 0.8
    alternative = greater
```

Here we can see that increasing the effect size has provided an even lower estimate for the sample size n1, and is thus still within the scope of our data collection.

References

[1] W. H. Press, *Numerical Recipes 3rd Edition: The Art of Scientific Computing*, Cambridge University Press, 2007.

[2] P. S. Addison, *The Illustrated Wavelet Transform Handbook: Introductory Theory and Applications in Science, Engineering, Medicine and Finance.* IOP Publishing, 2002.

[3] G. Kaiser, *A friendly guide to wavelets.* Birkhauser, Boston, 1994.

[4] A. Stefanovska, M. Bračič and H. D. Kvernmo. Wavelet analysis of oscillations in the peripheral blood circulation measured by laser Doppler technique, *IEEE Trans. Bio. Med. Eng.*, **46**,1230–1239, 1999.

[5] Y. Shiogai, A. Stefanovska and P. V. E. McClintock. Nonlinear dynamics of cardiovascular ageing, *Phys. Rep.*, **488**,51–110, 2010.

[6] P. Clemson and A. Stefanovska. Discerning non-autonomous dynamics, *Phys. Rep.*, 10.1016/j.physrep.2014.04.001, 2014

[7] J. L. Hodges Jr and E. L. Lehmann. The efficiency of some nonparametric competitors of the t-test, *Ann. Math. Statist.*, 324–335, 1956.

[8] L. M. Bachmann, M. A. Puhan, G. ter Riet and P. M. Bossuyt. Sample sizes of studies on diagnostic accuracy: literature survey, *BMJ*, **332**, 1127–1129, 2006.

[9] S. Jones, S. Carley and M. Harrison. An introduction to power and sample size estimation, *Emerg. Med. J.*, **20**, 453, 2003.

[10] S. Champely. Basic functions for power analysis, http://cran.r-project.org/web/packages/pwr/pwr.pdf, 2009.
